# Editorial: Acute toxicities and late effects of childhood cancer treatment

**DOI:** 10.3389/fped.2023.1335940

**Published:** 2023-12-05

**Authors:** Małgorzata Czogała, Katarzyna Muszyńska-Rosłan

**Affiliations:** ^1^ Department of Pediatric Oncology and Hematology, Institute of Pediatrics, Jagiellonian University Medical College, Krakow, Poland; ^2^ Department of Pediatric Oncology and Hematology, Medical University of Bialystok, Bialystok, Poland

**Keywords:** children, acute toxicities, late effects, cancer, treatment

**Editorial on the Research Topic** Acute toxicities and late effects of childhood cancer treatment

Treatment results in pediatric cancers have improved significantly during the last decades due to the development of intensive chemotherapy, radiotherapy, surgery, and novel therapeutic measures such as immunotherapy and other targeted treatment methods. In parallel, great progress was made in supportive care to manage the broad spectrum of acute toxicities affecting most organs in children during anticancer therapy. Most common acute toxicities are reversible, however, some are still life-threatening. Despite increasing awareness and improvement in long-term care of pediatric cancer survivors, late effects still affect the quality of life and increase mortality after anticancer therapy in childhood. Multidisciplinary collaboration between pediatricians of many specializations, specialists of internal medicine, psychologists, and physiotherapists is crucial to providing complete long-term care during childhood and adulthood for pediatric cancer survivors.

The present Research Topic on acute toxicities and late effects of childhood cancer treatment at Frontiers in Pediatrics encompasses three original studies, one consensus on toxicities definitions, and a single-case observation that addresses different aspects of childhood cancer treatment's acute and long-term effects.

Cheung et al. evaluated the associations of biomarkers of inflammation with functional outcomes in survivors of childhood acute lymphoblastic leukemia (ALL). Their findings suggested that systemic inflammation may be one of the important pathophysiological mechanisms underlying attention impairment and neurobehavioral symptoms in long-term survivors of pediatric ALL. The authors proposed that markers of inflammation could be used to assess or monitor the effectiveness of interventions in improving cognitive outcomes in survivors.

In the study by Brackmann et al., a self-administered questionnaire assessing childhood cancer treatment was evaluated. Moreover, the authors analyzed associations between exposure to cancer therapies and late effects. The study revealed that the questionnaire was reliable for retrospective assessment of exposure to chemotherapy and radiotherapy in childhood cancer survivors of secondary primary neoplasm, however, self-reported radiotherapy for survivors of first primary neoplasm was imprecise. The authors found an association between the use of chemotherapy and late effects such as hypercholesterolemia and thyroid diseases, excluding thyroid cancer.

Zaletel et al. described the results of screening for breast cancers in long-term survivors of childhood Hodgkin's lymphoma. The authors conducted a population-based study on 105 female patients treated for HL in Slovenia between 1966 and 2010. The study revealed a cumulative incidence of secondary breast cancer (SBC) of 15.2% at 40 years of follow-up after chest radiotherapy. As a result of the screening, all SBCs in the analyzed group were diagnosed at an early stage and there was no death due to SBC in the cohort. The authors concluded that regular and long-term follow-up with breast cancer screening and breast self-examination is of vital importance in patients treated with chest radiotherapy.

Bigagli et al. described a case of cytomegalovirus retinopathy and encephalopathy following high-dose thiotepa and proton irradiation in a pediatric patient with high-risk medulloblastoma. Cytomegalovirus infection confirmed with quantitative and qualitative blood PCR analysis manifested with complete blindness, radiological patterns of leukoencephalopathy and retinopathy, elevated liver enzymes, and persistent thrombocytopenia. Treatment with oral valganciclovir led to the normalization of liver function and negativization of blood CMV DNA, however, the blindness persisted.

Nielsen et al., on behalf of the Ponte di Legno Severe Toxicity Working Group, presented modified consensus definitions of 21 physician-defined severe toxicities (STs), proposed to be included in outcome reporting of cancer treatment. STs were selected and defined based on five criteria: not present before cancer diagnosis, symptomatic, objective, of unacceptable severity, and permanent or requiring unacceptable treatments. The modified ST consensus definitions enable statistical analyses of STs occurring during and after cancer treatment and uniform future reporting of STs across international cohorts as a part of cancer treatment evaluation. It will allow comparison of the toxicity patterns across different treatment protocols and research on risk factors of STs. Finally, it may lead to modifications in cancer therapy and early targeted interventions to further reduce toxicities without compromising cure.

The presented Research Topic certainly does not cover all issues related to early and late complications of cancer treatment in children. However, we hope that the published papers will contribute to a better understanding of the theme. We propose that increasing awareness of acute and long-term complications of cancer therapy in childhood will contribute to treatment modifications and finally improve the quality of life of pediatric cancer survivors.

